# Prediction of the aggressiveness of non-functional pancreatic neuroendocrine tumors based on the dual-tracer PET/CT

**DOI:** 10.1186/s13550-019-0585-7

**Published:** 2019-12-23

**Authors:** Susanna Majala, Hanna Seppänen, Jukka Kemppainen, Jari Sundström, Camilla Schalin-Jäntti, Risto Gullichsen, Jukka Schildt, Harri Mustonen, Tiina Vesterinen, Johanna Arola, Saila Kauhanen

**Affiliations:** 1 Division of Digestive Surgery and Urology, Turku University Hospital, P.O. Box 52, FIN-20521 Turku, Finland; 2 Turku PET Centre, Turku University Hospital, P.O. Box 52, FIN-20521 Turku, Finland; 3 Department of Abdominal Surgery, Translational Cancer Medicine Research Program, Faculty of Medicine, University of Helsinki and Helsinki University Hospital, P.O. Box 340, FIN-00029 Helsinki, Finland; 4 Department of Clinical Physiology and Nuclear Medicine, Turku University Hospital, P.O. Box 52, FIN-20521 Turku, Finland; 5 Department of Pathology, Turku University Hospital, P.O. Box 52, FIN-20521 Turku, Finland; 6 Department of Endocrinology, Abdominal Center, University of Helsinki and Helsinki University Hospital, P.O. Box 340, FIN-00029 Helsinki, Finland; 7 Department of Clinical Physiology and Nuclear Medicine, Helsinki University Hospital, Haartmaninkatu 4, FIN-00029 Helsinki, Finland; 8 Department of Surgery, University of Helsinki and Helsinki University Hospital, P.O. Box 340, FIN-00029 Helsinki, Finland; 9 HUSLAB, Department of Pathology, University of Helsinki and Helsinki University Hospital, P.O. Box 400, FIN-00029 Helsinki, Finland; 10 Institute for Molecular Medicine Finland (FIMM), HiLIFE, University of Helsinki, P.O. Box 20, FI-00014 Helsinki, Finland

**Keywords:** NF-PNET, ^18^F-FDG-PET/CT, ^68^Ga-DOTANOC-PET/CT, prospective study, surgical management, Ki-67

## Abstract

**Background:**

Predicting the aggressive behavior of non-functional pancreatic neuroendocrine tumors (NF-PNET) remains controversial. We wanted to explore, in a prospective setting, whether the diagnostic accuracy can be improved by dual-tracer functional imaging ^68^Ga-DOTANOC and ^18^F-FDG-PET/CT in patients with NF-PNETs.

**Methods:**

Thirty-one patients with NF-PNET (90% asymptomatic) underwent PET-imaging with ^18^F-FDG and ^68^Ga-DOTANOC, followed by surgery (*n* = 20), an endoscopic ultrasonography and fine-needle biopsy (*n* = 2) or follow-up (*n* = 9). A focal activity on PET/CT greater than the background that could not be identified as physiological activity was considered to indicate tumor tissue. The imaging results were compared to histopathology. The mean follow-up time was 31.3 months.

**Results:**

Thirty-one patients presented a total of 53 lesions (40 histologically confirmed) on PET/CT. Thirty patients had a ^68^Ga-DOTANOC-positive tumor (sensitivity 97%) and 10 patients had an ^18^F-FDG-positive tumor. In addition, one ^68^Ga-DOTANOC-negative patient was ^18^F-FDG-positive. ^18^F-FDG-PET/CT was positive in 19% (3/16) of the G1 tumors, 63% (5/8) of the G2 tumors and 1/1 of the well-differentiated G3 tumor. ^68^Ga-DOTANOC-PET/CT was positive in 94% of the G1 tumors, 100% of the G2 tumors and 1/1 of the well-differentiated G3 tumor. Two out of six (33%) of the patients with lymph node metastases (LN+) were ^18^F-FDG-positive. The ^18^F-FDG-PET/CT correlated with tumor Ki-67 (*P* = 0.021). Further, the Krenning score correlated with tumor Ki-67 (*P* = 0.013). ^18^F-FDG-positive tumors were significantly larger than the ^18^F-FDG-negative tumors (*P* = 0.012). ^18^F-FDG-PET/CT showed a positive predictive value of 78% in the detection of potentially aggressive tumors (G2, G3, or LN + PNETs); the negative predictive value was 69%.

**Conclusions:**

^18^F-FDG-PET/CT is useful to predict tumor grade but not the LN+ of NF-PNETs. Patients with ^18^F-FDG-avid NF-PNETs should be referred for surgery. The ^68^Ga-DOTANOC-PET/CT also has prognostic value since the Krenning score predicts the histopathological tumor grade.

**Trial registration:**

The study has been registered at ClinicalTrials.gov; Non-functional Pancreatic NET and PET imaging, NCT02621541.

## Background

Pancreatic neuroendocrine tumors (PNETs) constitute 3% of all pancreatic neoplasms and 60–80% of PNETs are defined as non-functional (NF-PNET) [1]. The incidence has increased in recent years due to the expanding use of imaging [2]. Despite the generally indolent nature, it has been recognized that the pathological potential of PNETs is highly variable and some NF-PNETs present at an advanced stage with local invasion and distant metastases [3, 4]. Heterogeneity of PNETs makes therapeutic decisions difficult with no clear consensus.

The most powerful prognostic factors are the grade and distant metastases [4]. Although complete surgical resection is the only potentially curative treatment, a conservative approach seems to be safe for asymptomatic and stable sporadic NF-PNET≤ 2 cm [5, 6]. However, a locally advanced disease with lymph node metastases (LN+) is rare but also possible on small (1–2 cm) NF-PNETs [7]. In a review of 136 surgical patients, Hashim and colleagues suggested a metastatic rate of 8% in PNETs as small as 1.5 cm [8]. In a retrospective analysis (*n* = 181), Partelli et al. [9] demonstrated that lymph node metastases decreased the 5-year disease-free survival in NF-PNETs (70% vs 97%, *P* < 0.001). Pancreatic resection is a high-risk operation with 20–40% morbidity and 1–2% mortality [10, 11]. Knowing the risks of pancreatic surgery, it is challenging to decide between surgery and follow-up for these patients.

The vast majority of well-differentiated PNETs express somatostatin receptors (SSTRs) and can be visualized by the binding of a radioactive somatostatin analog. SSTR-based functional imaging, ^68^Ga-DOTANOC positron emission tomography/computed tomography (PET/CT) has a high diagnostic sensitivity (88–100%) for localizing NETs [12–14] but provides limited information about the aggressiveness of PNET. ^18^F-fluoro-2-deoxyglucose (FDG)-PET/CT demonstrates increased cellular tissue metabolism. Although the sensitivity of ^18^F-FDG-PET/CT imaging is low for NETs, positive ^18^F-FDG-PET/CT predicts poor survival for NET patients [15, 16]. Dual-tracer functional imaging of NF-PNET has only been studied in retrospective series [17, 18].

The aim of this study was to determine the role of dual-tracer functional imaging in predicting aggressive behavior in NF-PNETs in a prospective setting with histopathological references. The hypothesis was that the higher the maximum standardized uptake value (SUV_max_) of ^18^F-FDG-PET/CT then the higher the Ki-67 of the tumor would be and the higher the SUV_max_ of ^68^Ga-DOTANOC-PET/CT the lower the Ki-67 of the tumor would be*.*

## Materials and methods

### Study design

The study was a prospective, multicenter clinical trial at Turku and Helsinki University Hospitals in Finland. From January 2016 to January 2018, a total of 35 patients suspected of having NF-PNET on a primary CT were prospectively imaged using ^68^Ga-DOTANOC-PET/CT and ^18^F-FDG-PET/CT. Four patients were excluded; one patient diagnosed with a pancreatic adenocarcinoma, another with a functional duodenal neuroendocrine tumor (gastrinoma), one with mixed neuroendocrine non-neuroendocrine neoplasm and one patient with an uncertain diagnosis lacking pathology. A total of 31 patients (age range 20-83 years; mean age 60 ± 18 years) were enrolled in the study. Patient characteristics are given in Table 1. Twenty patients were operated. Four patients underwent endoscopic ultrasonography and fine-needle biopsy (EUS-FNB), but two of these were found to be non-specific. One patient with Von Hippel-Lindau (VHL) syndrome had a pancreatic intraepithelial neoplasia lesion in the histopathological analysis and two NF-PNETs (Ø 13 and 10 mm on MRI) were followed up. Twenty-two patients had a histopathological confirmation (20 resection specimens and two EUS-FNB) and nine patients with a positive ^68^Ga-DOTANOC PET/CT finding were followed up (33.5 ± 6.2 months). Thirty-one patients had a total of 53 lesions on the PET/CT imaging. The median PET/CT imaging interval was 34 days (range 2–164, IQR 12–62 days) in 29 patients. The PET/CT imaging interval was delayed in two patients, in 1 to 360 days due to logistical reasons and in another to 244 days due to a more urgent operation. Since there were two methods to measure chromogranin A (CgA) in use at the laboratories during the study period, the CgA was reported as three subgroups. For an accurate diagnosis of PNETs every attempt was made to establish a tissue sample by means of an operation or an EUS-FNB. Follow-up time was measured from the date of the first PET/CT scan to the review time. Patients were treated in accordance with the routine procedures of the departments and the European Guidelines [19]. The study has been registered at ClinicalTrials.gov (NCT02621541).

**Table 1:** Patient characteristics

No. patients	31
Gender, Male, *n* (%)	20 (65)
Age years, mean (SD)	59.6 (17.6)
BMI, mean (SD)	26.3 (4.0)
Asymptomatic, *n* (%)	29 (90)
MEN1 syndrome, *n* (%)	7 (23)
CgA, *n* (%)	
Strongly positive	3 (10)
Weakly positive	15 (48)
Negative	13 (42)
PP (pmol/L), median (IQR)	68 (41–129)
5-HIAA (nmol/L), median (IQR)	64 (53–64)
Primary tumor size (mm)	
All patients, median (IQR)	24 (14–35)
Operated, median (IQR) (*n* = 20)	31 (20–52)
Biopsy and follow-up, median (min–max) (*n* = 2)	24 (22–25)
Follow-up*, median (IQR) (*n* = 9)	14 (13–21)
Tumor localization, *n* (%)	
Head	12 (39)
Body	2 (6)
Tail	10 (32)
Multiple	7 (23)
Treatment, *n* (%)	
Surgery	20 (65)
Biopsy and follow-up	2 (6)
Follow-up*	9 (29)
Type of surgery, *n* (%)	
Total pancreatectomy	2 (10)
Pancreaticoduodenectomy	4 (20)
Distal pancreatectomy	13 (65)
Enucleation	1 (5)
Type of surgery, *n* (%)	
Open	13 (75)
Laparoscopic	5 (25)
Robotic surgery	2 (10)
Grade, *n* (%)	
G1	13 (59)
G2	8 (36)
G3 NET	1 (5)
G3 NEC	0

Abbreviations: *CgA*, circulating chromogranin A, strongly positive indicates S-CgA = 13.5 nmol/L or P-CgA 9–37 nmol/L, weakly positive indicates S-CgA 2.2–4.7 nmol/L or P-CgA 3.0–4.8 nmol/L and negative indicates S-CgA < 2.1 nmol/L or P-CgA < 3.0 nmol/L; *BMI*, body mass index, kg/m^2^; *MEN1*, multiple endocrine neoplasia type 1 syndrome; *PP*, pancreatic polypeptide; *5-HIAA*, 5-hydroxyindoleatic acid

*Two patients underwent non-diagnostic EUS-FNB (endoscopic ultrasonography and fine needle biopsy)

### ^68^Ga-DOTANOC and ^18^F-FDG-PET/CT protocol

PET/CT was performed using the Discovery STE (6 ^68^Ga-DOTANOC and 14 ^18^F-FDG-PET/CT) or VCT (11 ^68^Ga-DOTANOC and 10 ^18^F-FDG-PET/CT) scanner (General Electric Medical Systems, Milwaukee, WI) and one patient underwent ^18^F-FDG-PET/(magnetic resonance imaging) MRI using an Ingenuity TF PET/MRI scanner (Phillips Medical Systems, Cleveland, OH) at Turku PET center and Siemens Biograph mCT 64 (Siemens Healthineers, Erlangen, Germany) (12 ^68^Ga-DOTANOC and 4 ^18^F-FDG-PET/CT) or Gemini PET-CT scanner (Philips Inc, USA) (1 ^68^Ga-DOTANOC and 1 ^18^F-FDG-PET/CT) at Nuclear Medicine Department in Helsinki University Hospital. One ^68^Ga-DOTANOC and 1 ^18^F- FDG-PET/CT scan were done at a private institute using the Siemens Biograph 6 (Siemens Medical Solutions, USA).

Patients underwent a whole-body PET/CT scan from the level of the skull base to the mid-thigh starting 64 ± 13 min after the injection of ^68^Ga-DOTANOC and 55 ± 9 min after ^18^F-FDG. The mean dose of intravenous ^68^Ga-DOTANOC was 143.8 ± 17.1 MBq and ^18^F-FDG was 321.9 ± 67.3 MBq. The patients fasted for 6 h before the study. Blood glucose levels were checked before any ^18^F-FDG-PET/CT for patients with diabetes or previous history of glucocorticoids use (range 4.6–8.3 mmol/l). A low-dose PET/CT was followed with a whole-body diagnostic CT scan after automated intravenous injection of the contrast agent, either with ^68^Ga-DOTANOC- or ^18^F-FDG-PET/CT. Attenuation correction was performed using a low-dose ultrafast CT protocol (80 mAs, 140 kV, 0.3 mSv per field of view). Images were reconstructed full width at half maximum and fully three-dimensional maximum-likelihood ordered-subset expectation maximization (OSEM). Data was corrected for dead time, decay, and photon attenuation and was reconstructed to a 128 × 128 matrix.

### Data analysis

**Fig. 1:**
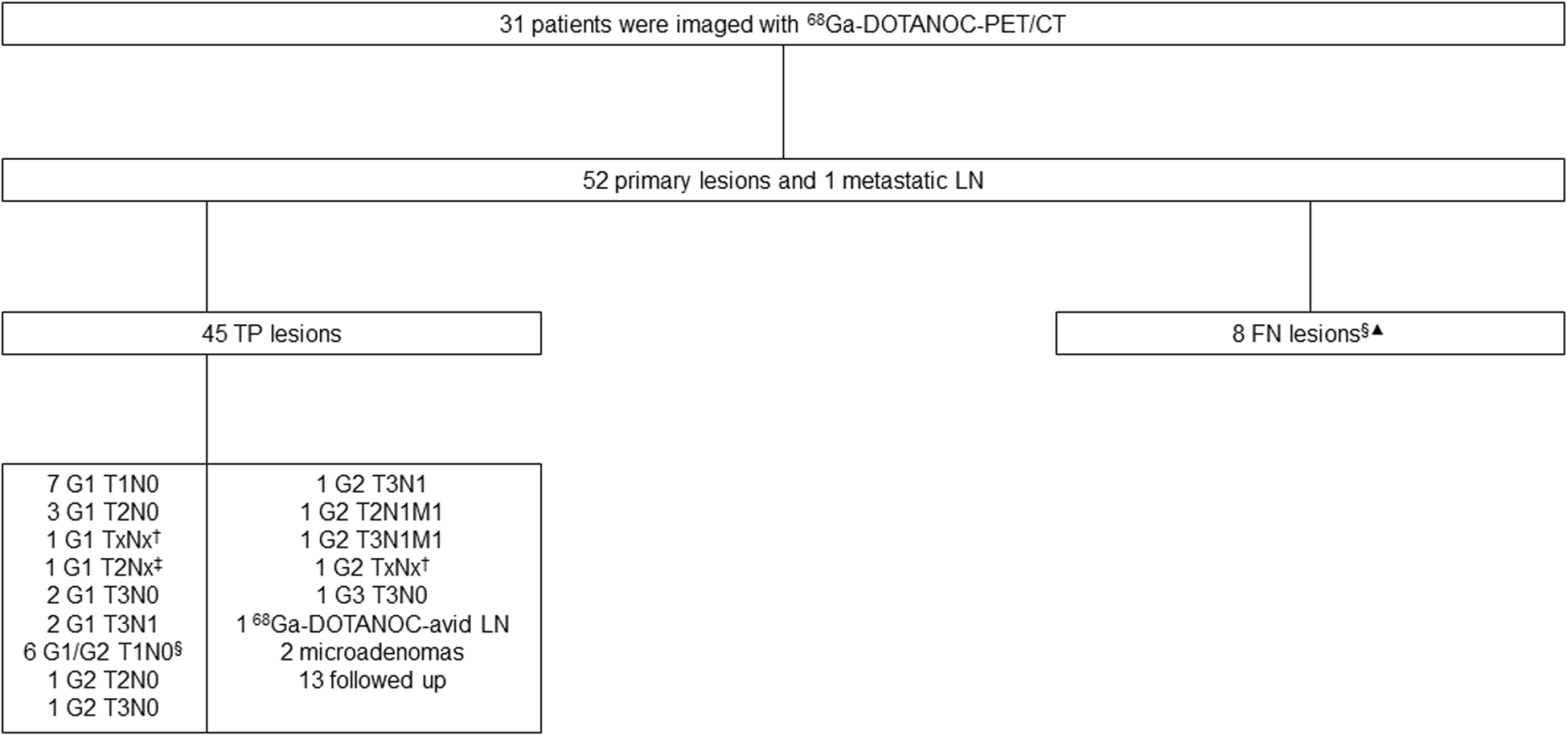
Characteristics of lesions detected on ^68^Ga-DOTANOC-PET/CT. TP, true-positive; FP, false-positive; TN, true-negative; FN, false negative; LN, lymph node. ^†^Diagnosis was made by biopsy, stage unknown. ^‡^Unknown lymph node status due to enucleation. ^§^MEN1 patient, who underwent total pancreatectomy: a total of 13 tumors (8 = G1, 5 = G2); 6 were detected on PET/CT (TP) and 7 were FN. ^▲^A ^18^F-FDG-avid G1 tumor with LN metastases

**Fig. 2:**
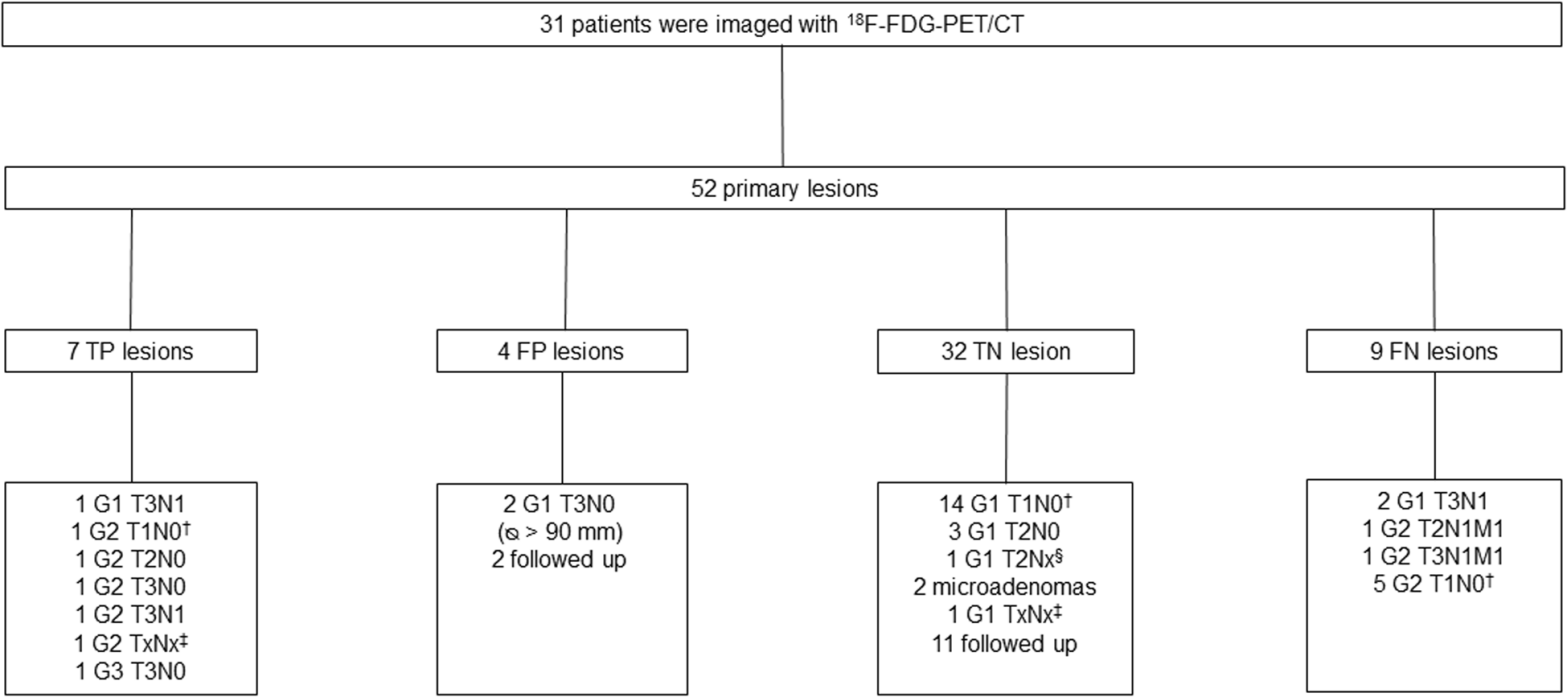
Characteristics of lesions on ^18^F-FDG-PET/CT. TP, true-positive, G2, G3, lymph node metastasis (LN+) or distant metastasis (M+); FP, false-positive, G1, no lymph node metastases (LN-) or no distant metastases (M-); TN, true-negative, : G1, LN- or M- ; FN, false negative, G2, G3, LN+ or M+. ^†^MEN1 patient underwent total pancreatectomy: on histopathological report total of 13 tumors was detected: (8 = G1, 5 = G2); 1 was ^18^F-FDG-positive (1 TP), and other 12 ^18^F-FDG-negative (8 TN and 4 FN). ^‡^Diagnosis was made by biopsy, stage unknown. ^§^Unknown LN status due to enucleation

The diagnostic accuracy of the PET/CT studies was assessed by comparing the PET-images and the histopathological reports (*n* = 22). When proper histology was not available (n=9), the consensus was based on the sum of the laboratory tests and imaging procedures. This information was used for the interpretation of the lesion analysis (Figs. 1 and 2). Data on the primary tumor site and the diameter were collected from the pathology reports and from other imaging studies (contrast CT or MRI). The histopathological analysis was blind and conducted by two experienced pathologist (J.A and J.Su). Clinical TNM and grade classification was based on the 2017 World Health Organization (WHO) classification of PNETs [5].

Only the patients and the tumors with a histopathological confirmation were included in the correlation analysis. Ki-67-labeling indexes were studied from the whole series of tumors using MIB-I antibody (Dako, Agilent Pathology Solutions, Santa Clara, CA, USA) and automated staining instrument (BenchMark Ultra, Ventana Medical Systems, Inc., Tucson, AZ, USA). All stainings were prepared in the clinical pathology laboratory (HUSLAB, Helsinki University Hospital) under standardized conditions. Stained slides were digitized with a Panoramic scanner (3DHISTECH, Budapest, Hungary) and Ki-67 values were calculated by T.V from hot spot areas comprising of at least 2000 cells. A publicly available application that was shown suitable for pancreatic NETs, was used for quantitative image analysis (ImmunoRatio) [20, 21].

Analyses of PET/CT-images were interpreted by a dedicated nuclear medicine physician (J.K), with short referral information but blinded to histopathological report. The SUV_max_–values were determined for every tumor or abnormal anatomical region on both ^68^Ga-DOTANOC and ^18^F-FDG-PET/CT. For the PET/CT-studies areas with a focal activity greater than the background that could not be identified as physiological activity were considered to indicate tumor tissue. Lesions were graded on ^68^Ga-DOTANOC-PET/CT with the Krenning score, by semi-quantitatively comparing SUV_max_ of the tumors to the reference organs such as liver and spleen. Krenning scoring was performed as follows: score 1: uptake < normal liver, 2: uptake = normal liver, 3: uptake > normal liver and 4: uptake > spleen or kidneys [22]. Further, by using the dual-tracer PET/CT the NETPET-score (grades P1–5) was defined. Grade P1 indicated purely somatostatin analog avid lesion without ^18^F-FDG-uptake and P5 indicated the presence of a significant ^18^F-FDG-positive and somatostatin analog-negative diseas e[23]. The imaging analysis was performed using ADW 4.4 workstation.

### Statistical analysis

Normally distributed variables were expressed as means and standard deviations (SD) variables not following a normal distribution as medians and interquartile ranges (IQR) and categorical variables as frequencies and proportions. The Shapiro-Wilk test was used to test deviations from a normal distribution. Spearman’s rank correlation was used to test the relationship between Ki-67 and SUV_max_ due to a lack of normally distributed data. The Mann-Whitney or the Kruskal-Wallis tests were used to discover the differences between the groups in continuous variables; Fisher’s exact test was used for binary variables and the linear by linear association test for ordinal variables. A *P* value of <0.05 was considered statistically significant, and two-tailed tests were used. The data analysis was performed using commercially available software (Statistical Package for Social Sciences, version 24, IBM Corp., Armonk, NY, USA).

## Results

### Features of NF-PNETs and histopathological grading

The type of surgery and histopathological finding of the tumors are presented in Table 1. Patients with G1 NF-PNET were more commonly asymptomatic (92%) compared to G2 patients (75% asymptomatic), but the only patient with a G3 tumor was also asymptomatic (*P* = 0.595). One symptomatic patient had jaundice and two had upper abdominal pains. All the symptomatic patients had sporadic NF-PNET.

Multiple endocrine neoplasia 1 (MEN1) patients (*n* = 7) more commonly had a G1 (71%) rather than a G2 (29%) tumor and none had a G3 tumor, but the difference was not statistically significant (*P* = 0.470). There was no correlation between the tumor size and the grade: G1 (34 ± 27 mm), G2 (36 ± 28 mm), and G3 (*n* = 1, 45 mm) (*P* = 0.800). However, all the operated primary tumors ≤ 2 cm (*n* = 6) were G1 tumors without lymph node metastases. The most common location for the G1 tumor was the tail of the pancreas (54%). Three out of eight (38%) of the G2 NF-PNETs were located in the head and the same amount; 3/8 (38%) in the tail of the pancreas. The G3 NF-PNET was located in the head of the pancreas. There was no statistical significance between the location of a tumor and the grade of a tumor (*P* = 0.554). There was a trend between the grade and CgA (*P* = 0.108).

### Correlation between ^68^Ga-DOTANOC-PET/CT and grade

^68^Ga-DOTANOC-PET/CT was positive in 30 patients and thus the sensitivity of the ^68^Ga-DOTANOC-PET/CT detecting of NF-PNETs was 97% and specificity was 100%. The only ^68^Ga-DOTANOC-negative tumor (G1) was ^18^F-FDG-positive and the patient had multiple (11/13) lymph node metastases. Thirty-one study patients had a total of 53 lesions, 45 of these were ^68^Ga-DOTANOC-positive (32/45 histologically confirmed lesions). Eight lesions were not detected by PET/CT at all and were false negative (FN) lesions (Fig. 1); one patient had negative ^68^Ga-DOTANOC-PET/CT and histologically confirmed G1 NET and seven FN lesions were detected on the same MEN1 patient who underwent a total pancreatectomy. He had a total of 13 PNETs (five G2 T1N0 tumors and eight G1 T1N0 tumors). Six of these lesions were detected on ^68^Ga-DOTANOC-PET/CT and one of these tumors was also ^18^F-FDG-positive. Three lesions were > 10 mm and 10 were 5–10 mm in size. There were also multiple microadenomas (NET< 5 mm). Due to several lesions, it was impossible to combine the information concerning the 13 tumors on a histopathological report and the six lesions on ^68^Ga-DOTANOC-PET/CT. Only the largest primary tumor was included in the lesion analysis.

**Fig. 3:**
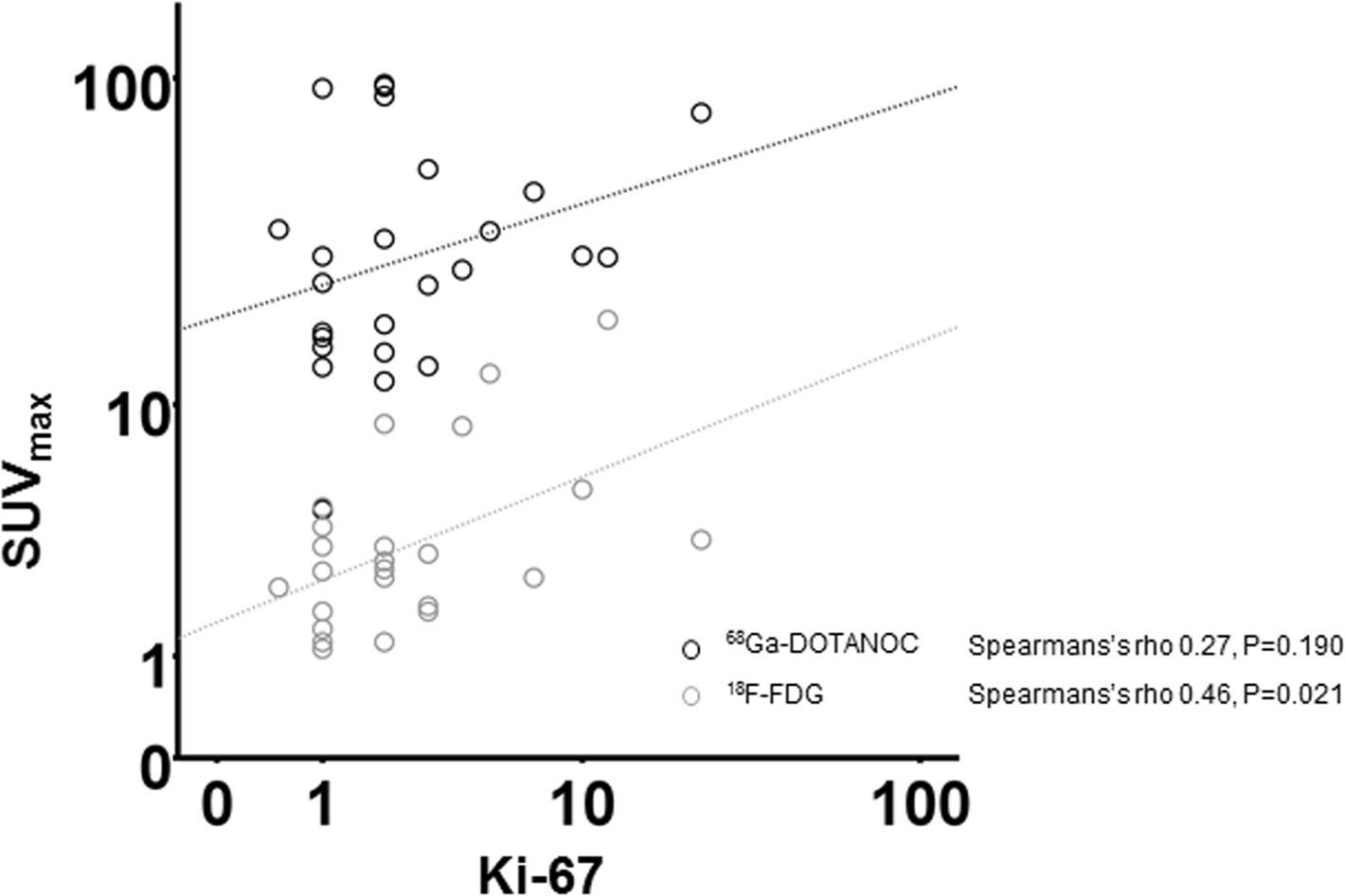
Logarithm variation of the relationship between the tumor Ki-67 and uptake of ^68^Ga-DOTANOC (black circles) and ^18^F-FDG (grey circles) for every tumor

The lesion-based sensitivity of ^68^Ga-DOTANOC-PET/CT to detect of NF-PNETs was 85% with a specificity of 100%. There was no correlation between the SUV_max_ of ^68^Ga-DOTANOC-PET/CT and the tumor Ki-67 (Spearmans’s ρ = 0.271, *P* = 0.190) (Fig. 3). We also analyzed the Krenning and NETPET score of the lesions (Table 2). There was a statistically significant correlation between both Krenning score and Ki-67 (*P* = 0.013) and NETPET score and Ki-67 (*P* = 0.036) (*n* = 43).

**Table 2:** Krenning and NETPET score of the lesions (*n* = 43)

Krenning score	*n* (%)
1	1 (2)
2	4 (9)
3	24 (56)
4	14 (33)
NETPET score	*n* (%)
P1	32 (75)
P2	8 (19)
P3	1 (2)
P4	1 (2)
P5	1 (2)

From 53 lesions, 43 were analyzed. Two microadenomas, 1 ^68^Ga-DOTANOC-positive lymph node, and 7 ^68^Ga-DOTANOC-negative false negative lesions of a patient with 13 NETs were not included to this analysis

### Correlation between ^18^F-FDG-PET/CT and grade

**Fig. 4:**
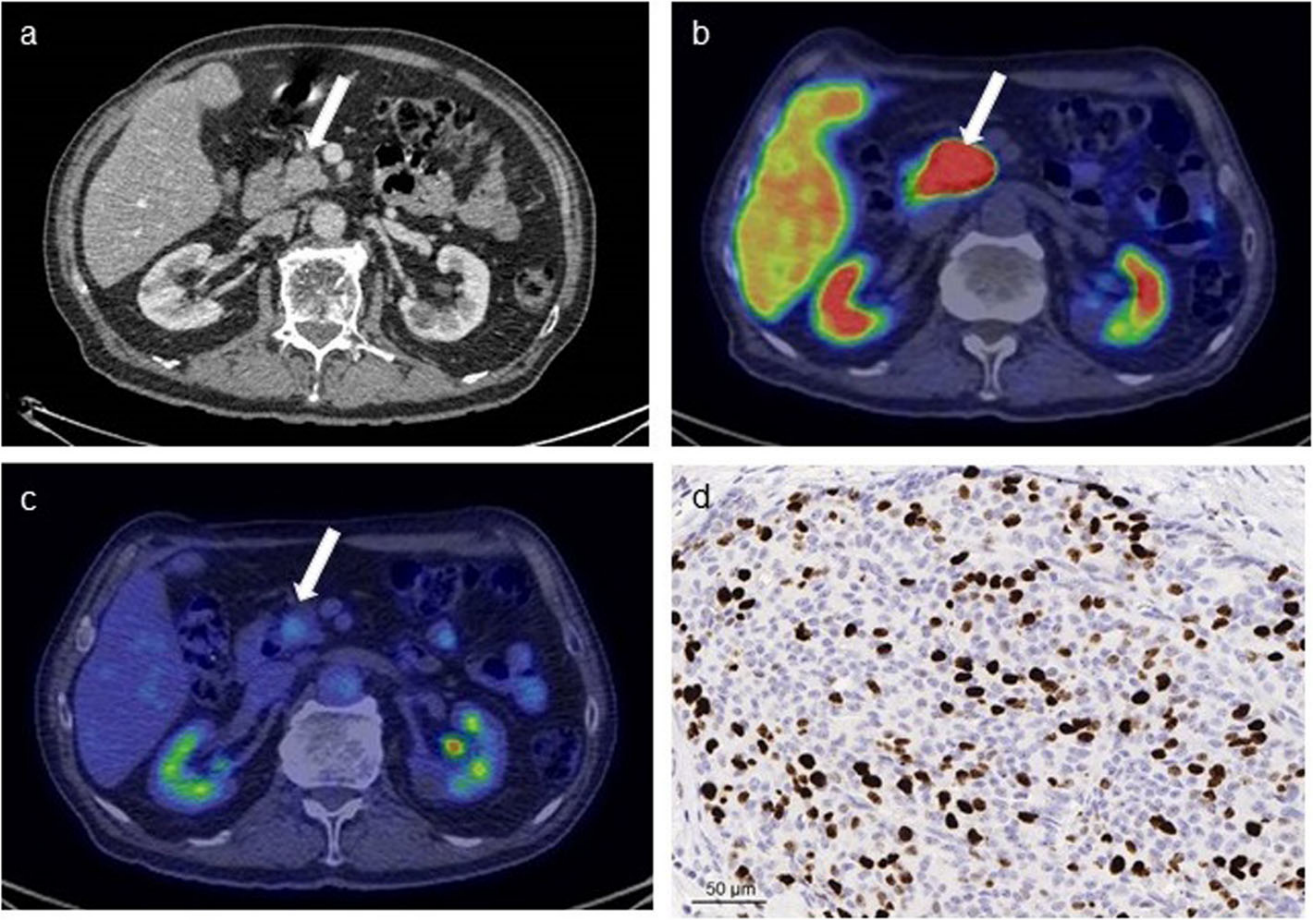
An 80-year-old male patient had a 3 cm solid mass in the head of the atrophic pancreas on CT (**a**). ^68^Ga-DOTANOC-PET/CT (**b**) showed intense uptake (SUV_max_ 79 g/ml) and ^18^F-FDG-PET/CT (**c**) was positive (SUV_max_ 3.4 g/ml, higher than liver background). He underwent a pancreaticoduodenectomy and histopathological analysis revealed a well-differentiated G3 PNET (Ki-67 31 %) (**d**, image magnification × 40) without lymph node metastases

^18^F-FDG-PET/CT was positive for 11 of 31 patients (35%) and the SUV_max_ ranged from 3.0 to 18.6 (median 4.5, IQR 3.4–8.7). Clinical characteristics of patients according to ^18^F-FDG-uptake are summarized in Table 3 and lesion characteristics are detailed in Fig. 2. In the lesion based analysis on ^18^F-FDG-PET/CT, 52 primary lesions were analyzed on 31 patients (Fig. 2). Three of the ^18^F-FDG-avid tumors were G1 (two T3N0 Ø ≥ 9 cm and one T3N1), five G2 (one T1N0, one T2N0, one T3N0, one T3N1 and one patient assessed by EUS-FNB, too frail for surgery) and one well-differentiated G3 (T3N0) tumor (Fig. 4). Two ^18^F-FDG-positive tumors are intensively followed up without a histological confirmation. ^18^F-FDG-PET/CT was positive in 19% (3/16) of G1 tumors, 63% (5/8) of G2 tumors and 100% (1/1) of G3 tumors. There was a statistically significant positive correlation between ^18^F-FDG-uptake and the tumor Ki-67 (Spearmans’s ρ = 0.458, *P* = 0.021) (Fig. 3). When tumors were divided according to ^18^F-FDG-positivity and ^18^F-FDG-negativity, the correlation with the grade stayed significant (*P* = 0.023) (Table 3). ^18^F-FDG-positive tumors were significantly larger than ^18^F-FDG-negative tumors, with a mean maximum diameter of 46 mm (median 33, IQR 23–90 mm) compared with 24 mm (median 20, IQR 12–30 mm; *P* = 0.012). All the ^18^F-FDG-negative patients were asymptomatic and 73% ^18^F-FDG-positive patients were asymptomatic (*P* = 0.037). The symptomatic patients had more intense ^18^F-FDG-uptake than the asymptomatic patients (*P* = 0.003). However, there was no significant correlation between symptoms and grade (*P* = 0.595) or symptoms and Ki-67 (*P* = 0.102). ^18^F-FDG-PET/CT showed a positive predictive value (PPV) of 78% in the detection of potentially aggressive tumors (G2, G3, LN + or M+ histologically confirmed PNETs) and the negative predictive value (NPV) was 69%.

**Table 3:** Clinical, histopathological and metabolic features of NF-PNETs

	^18^F-FDG-positive SUV_max_ range 3.0–8.6	^18^F-FDG-negative SUV_max_ range 0.8–3.2	*P*	^68^Ga-DOTANOC-positive SUV_max_ range 8.7–104.7	^68^Ga-DOTANOC-negative SUV_max_ 4.4	*P*
Sex			1.000			1.000
Male	7/11 (64)	13/20 (65)		19/30	1/1	
Female	4/11 (36)	7/20 (35)		11/30	0/1	
Age, years	67 (38–78)	68 (50–71)	1.000	66 (47–72)	70	0.903
Primary tumor size, mm	33 (23–90)	20 (13–30)	*0.023*	24 (14-38)	33	0.581
Asymptomatic	8/11 (73)	20/20 (100)	*0.037*	27/30	1/1	1.000
CgA			0.258			0.516
Strongly positive	2/11 (18)	1/20 (5)		3/30	0/1	
Weakly positive	6/11 (55)	9/20 (45)		15/30	0/1	
Negative	3/11 (27)	11/20 (50)		12/30	1/1	
Location			0.630			1.000
Head	6/11 (55)	6/20 (30)		11/30	1/1	
Body	0/11 (0)	2/20 (10)		2/30	0/1	
Tail	3/11 (27)	7/20 (35)		10/30	0/1	
Multifocal	2/11 (18)	5/20 (25)		7/30	0/1	
Grade*			*0.023*			1.000
G1	3/9 (33)	13/16 (81)		15/24	1/1	
G2	5/9 (56)	3/16 (19)		8/24	0/1	
G3	1/9 (11)	0/16 (0)		1/24	0/1	
Ki-67	4.0 (1.5–11.0)	2.0 (1.0–2.5)	0.051	2.0 (1.0–4.5)	1.0	0.384
^68^Ga-DOTANOC-positive	10/11 (91%)	20/20 (100%)	0.355			
^18^FDG-positive				10/30 (33%)	1/1 (100%)	0.355
LN+^‰^	2/8 (33)	4/11 (36)	1.000	5/30	1/1	0.316

Categorical variables are expressed as *n* (%) and continuous variables are reported as median values (IQR)

Abbreviations: *CgA*, circulating chromogranin A, Strongly positive indicates S-CgA = 13.5 nmol/L or P-CgA 9–37 nmol/L, weakly positive indicates S-CgA 2.2–4.7 nmol/L or P-CgA 3.0–4.8 nmol/L and negative indicates S-CgA < 2.1 nmol/L or P-CgA < 3.0 nmol/L; *LN+*, lymph node metastases

*Lesion analysis (histologically confirmed, *n* = 25)

^‰^Follow-up patients, biopsied patients and one patient who underwent enucleation were excluded because it was not possible to assess their LN+

### Local and distant metastases

**Fig. 5:**
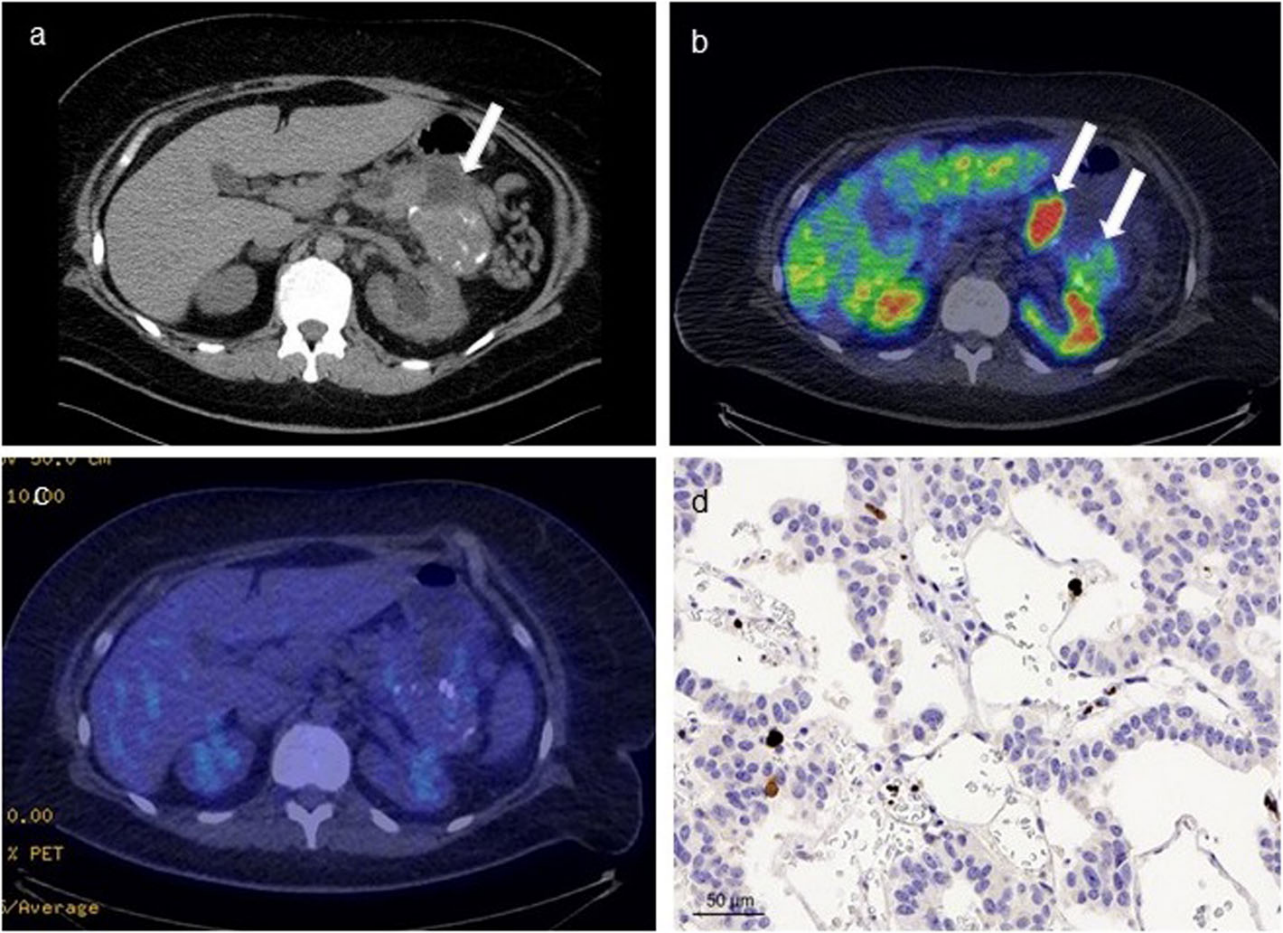
A 32-year-old female patient had a 10 cm complex cystic-solid mass in the tail of the pancreas on CT (**a**). ^68^Ga-DOTANOC-PET/CT (**b**) showed two different uptake intensity areas around a large cystic component (SUV_max_ 29 g/ml and 10.6 g/ml) in the pancreatic tumor. ^18^F-FDG-PET/CT (**c**) was negative (SUV_max_ 3.2 g/ml). She underwent a distal pancreatectomy and splenectomy and histopathological analysis revealed a G1 PNET (Ki-67 < 2%) (**d**, image magnification × 40) with five lymph node metastases

Six patients had histopathologically confirmed lymph node metastases (mean 4.8 ± 3.4 nodes). Three LN+ were in patients with G1 NF-PNETs and three in patients with G2 NF-PNETs. All the LN+ patients were asymptomatic and sporadic. Two of these six patients with LN+ (33%) had an ^18^F-FDG-positive tumor and four tumors with LN+ were ^18^F-FDG-negative (67%). Thus, 18% (2/11) of ^18^F-FDG-patients had LN+ vs. 20% (4/20) of ^18^F-FDG-negative patients had LN+ (Fig. 5). In patients with LN+, the mean primary tumor size was 54 ± 25 mm (range 24–90 mm). In five patients, preoperative ^68^Ga-DOTANOC-PET/CT did not reveal LN metastases. One patient had several LN metastases but ^68^Ga-DOTANOC-PET/CT showed only one metastatic lesion. Two patients were diagnosed with a NF-PNET in the tail of the pancreas and liver metastasis (T3N1M1/G2/Ki-67 10% and T2N1M1/G2/Ki-67 3%). Liver resection is arranged for another patient but to another no liver resection was performed due to progression of the disease.

### Prognosis and PET outcome

During the mean follow-up of 31.3 ± 6.2 months, one patient died (due to long-term surgical complications). He had a ^68^Ga-DOTANOC-negative and ^18^F-FDG-positive G1 T3N1 tumor. No recurrence had been detected during the follow-up time. One patient with ^68^Ga-DOTANOC-positive and ^18^F-FDG-negative primary tumor and liver metastases underwent a laparoscopic distal pancreatectomy and splenectomy. Histopathological analysis confirmed a G2 T3N1M1 tumor. Due to the progression of the disease no liver resection was arranged and capecitabine and temozolomide combination therapy and previously Lutetium-177-octreotate therapy was performed. One MEN1 patient with ^68^Ga-DOTANOC-positive and ^18^F-FDG-negative primary tumor underwent distal pancreatectomy and splenectomy and histological analysis revealed a G2 T2N1M1 tumor. Currently, a liver resection is planned due to the residual liver metastasis. One patient underwent robotic distal pancreatectomy and splenectomy due to two sporadic T1N0 G1 tumors. Stable residual tumor at the body of the pancreas is followed up. Among the follow-up group the VHL patient’s tumor has enlarged and uptake of ^18^F-FDG increased and surgery is considered. No disease progression has been detected in other patients belonging to the follow-up group (9 patients, 11 lesions).

## Discussion

To our knowledge, this is the first prospective study evaluating the impact of combined ^68^Ga-DOTANOC and ^18^F-FDG-PET/CT in predicting the aggressive behavior of NF-PNET. There is some previous data of the dual-tracer imaging with series of different gastroenteropancreatic neuroendocrine tumors [18, 24] and retrospective series of PNETs [17, 25]. The main interest of this study was to evaluate if the malignant potential of NF-PNETs could be predicted with dual-tracer PET/CT imaging. We found that there was a statistically significant positive correlation between ^18^F-FDG-uptake and the proliferation index of the tumor (*P* = 0.021). However, there was no negative correlation between ^68^Ga-DOTANOC-uptake and the Ki-67-index (*P* = 0.190). Both the Krenning score and NETPET score correlated statistically significantly with the Ki-67. In this patient cohort, the lower ^68^Ga-DOTANOC uptake, expressed as SUV_max_ values, was not directly associated to more aggressive phenotype unless the comparison was made to physiological organ activity by using Krenning score or combined to ^18^F-FDG-imaging in NETPET score. We also studied the ability of the dual-tracer PET/CT to assess LN+ but no effect was seen in order to enable a diagnosis of a local metastatic disease.

^18^F-FDG-PET/CT imaging has traditionally been used to assess adenocarcinoma and poorly-differentiated G3 neuroendocrine carcinomas (NECs). However, recent data has shown that ^18^F-FDG-PET/CT has prognostic value in patients with well-differentiated NET, showing that high uptake is associated with increased risk of early progression [5, 26]. In retrospective series the prognostic value of ^18^F-FDG-PET/CT imaging was even more powerful than the grade of the tumor [15, 16]. A study by Chan et al. [23] of 62 metastatic NET patients combined the results of somatostatin analogue and ^18^F-FDG-PET/CT to obtain an imaging biomarker, a NETPET-score (grades P1–5), and a classification system reflecting the burden of the disease. Grade P1 indicated purely somatostatin analogue avid lesion without ^18^F-FDG-uptake and P5 indicated the presence of a significant ^18^F-FDG-positive and somatostatin analogue-negative disease. The NETPET-score correlated positively with the histopathological grade and offered a better prediction of overall survival. In concordance with this we found a positive correlation between the NETPET score and the proliferation index. Further there was a correlation between the SUV_max_ of ^18^F-FDG-PET/CT and the proliferation index, and this correlation maintained significance when the patients were divided into WHO grades by Ki-67. Sharma et al. [27] concluded that SUV_max_ on ^68^Ga-DOTANOC-PET/CT was an independent, positive prognostic factor in patients with well-differentiated NET in a two-year median follow-up time. However, in light of this observation, we did not find significant prognostic role for ^68^Ga-DOTANOC-uptake in our study due to short follow-up time.

SSTR-based functional imaging with ^68^Ga-labelled peptides, ^68^Ga-DOTATATE, ^68^Ga-DOTATOC, and ^68^Ga-DOTANOC, has recently become the gold standard in the diagnosis of the NETs due to better sensitivity compared to ^111^In-DTPA-octreotide (Octreoscan®) [28] and lower patient radiation and higher spatial resolution than SPECT/CT [29]. ^68^Ga-DOTATATE has the strongest SSTR2-binding affinity. However, ^68^Ga-DOTATOC and especially ^68^Ga-DOTANOC have wider affinity profiles, including SSTR2, SSTR3 and SSTR5. The three analogs have shown no differences in clinical practice.

The sensitivity of the ^68^Ga-DOTANOC PET/CT for detecting NF-PNETs is high and SSTR-based imaging is a mandatory procedure to guide treatment planning [30]. In our series, the sensitivity was 97% and the smallest ^68^Ga-DOTANOC-avid and histologically confirmed tumor was 6 mm. Lesion-based analysis sensitivity was 85% (45/53). Seven FN lesions occurred in the same MEN1 patient who had a total of 13 PNETs (smallest 5–10 mm in size) in the histopathological analysis. The fact that not all of these multiple lesions of this MEN1 patient were detected with the ^68^Ga-DOTANOC should not be considered a diagnostic failure.

The prognostic value of ^18^F-FDG-PET/CT in functional imaging of NETs has been a target for recent research. Garin et al. [24] investigated prospectively well-differentiated metastatic NETs and reported that ^18^F-FDG-positivity correlated with decreased progression free survival and overall survival. Cingarlini et al. [17] compared retrospectively G1 and G2 PNETs and concluded that ^18^F-FDG-PET/CT was positive in a smaller proportion of G1 tumors (20%) compared with G2 tumors (76%)*.* In our study 34% NF-PNET patients had a positive ^18^F-FDG-PET/CT and the ^18^F-FDG-PET/CT showed a PPV of 78% in the detection of potentially aggressive tumors (G2, G3, LN+ or M+ PNETs), and the NPV was 69%. ^18^F-FDG-PET/CT should have some relevance when treatment options are discussed but the possibility of false imaging findings, especially false negative, should be kept in mind. In this prospective study there was a statistically significant correlation between the SUV_max_ of ^18^F-FDG-PET/CT and the Ki-67 of the primary tumor.

In the diagnostic workup of NF-PNETs, an endoscopic ultrasonography-guided fine-needle aspiration (EUS-FNA) could be an option, but it does not necessarily represent the whole tumor in terms of aggressiveness and the tissue material may be insufficient. In the French study [31], 30% of tumor grading was up-scaled on the resected tissue. Additionally, the heterogeneity in the tumor tissue may interfere the assessment of Ki-67-labeling [32]. However, in the prospective, multicenter, randomized controlled trial the EUS-FNB, which also was used in our study, produced a more accurate diagnosis than EUS-FNA [33].

Boninsegna et al. [34] found that Ki-67 > 5% and the ratio between the number of metastatic LNs above that of the examined LNs were better predictors of the recurrence than tumor size. Further, in our series there was no correlation between the tumor size and Ki-67-index, but the ^18^F-FDG-positive tumors were significantly larger than the ^18^F-FDG-negative tumors.

Our study results show that dual-tracer PET/CT imaging has a complementary role and it enables the detection of a potentially aggressive disease during the diagnostic work-up on well-differentiated NF-PNETs. Due to this we recommend ^18^F-FDG-PET/CT to be a part of a systematic diagnostic work-up of asymptomatic NF-PNETs. Especially if a consensus regarding surgical therapy cannot be reached, dual-tracer PET/CT could supplement the evaluation of the nature of the tumor. Due to our results ^18^F-FDG-avid tumors should be considered for surgery if there are no contraindications. In a case of a ^18^F-FDG-negative lesion follow-up could be considered if all the other aspects of the diagnostic work-up also support this treatment strategy.

The strengths of the present study were histopathological re-evaluation of all the tumors as well as the immunohistochemical Ki-67 staining and automated image analysis. The major limitation of the present study is the small number of patients, as the disease is rare. In addition, the lack of histopathological confirmation of the follow-up patients limits the analysis. For this subgroup of patients, the collection of samples was not technically or ethically feasible. However, the correlation analyses were performed with histopathologically confirmed cases. Since pancreatic neuroendocrine carcinomas are rare, the study population consists of well-differentiated PNETs, which impairs the sensitivity and specificity of ^18^F-FDG-PET/CT to detect potentially aggressive tumors. In two patients the interval between PET imaging was delayed (244 and 360 d), however, due to the indolent nature of NF-PNETs the long dual PET/CT interval is likely to be of no great importance and we preferred not to exclude these patients. Another limitation is the relatively short follow-up time (mean 31.3 mo, SD 6.2 mo) which is insufficient to estimate a definitive prognosis. There is a possibility that during a longer follow-up time the ^18^F-FDG-positive tumors in this study would prove to be progressive, and our intent is to analyze long time prognosis. In addition, it would be essential to detect which biological and immunohistochemical features are common for these tumors.

## Conclusion

SUV_max_ measured on ^18^F-FDG-PET/CT was significantly associated with the proliferation index, Ki-67, and will serve as a surrogate measure of tumor aggressiveness in patients with asymptomatic NF-PNET. Further, the ^68^Ga-DOTANOC-PET/CT has notable prognostic value since the Krenning score predicts the histopathological tumor grade.

To conclude, we recommend ^18^F-FDG-PET/CT to be a part of a systematic diagnostic work-up of asymptomatic NF-PNETs. This prospective study shows that dual-tracer PET/CT imaging, using ^18^Ga-DOTANOC and ^18^F-FDG, has a complementary role and it enables the detection of a potentially aggressive disease during the diagnostic work-up on well-differentiated NF-PNETs.

## Data Availability

The datasets used and/or analyzed during the current study are available from the corresponding author on reasonable request.

## Abbreviations

^18^F-FDG: ^18^F-fluoro-2-deoxyglucose
5-HIAA: 5-hydroxyindoleatic acid
^68^Ga-DOTANOC: ^68^Ga-DOTA-1-Nal^3^-octreotide
^68^Ga-DOTATATE: ^68^Ga-DOTA-Tyr^3^-octreotate
^68^Ga-DOTATOC: ^68^Ga-DOTA-Tyr^3^-octreotide
ADW: Advantage Workstation for Diagnostic Imaging
BMI: Body mass index
Bq: Becquerel
CgA: Chromogranin A
CT: Computed tomography
EUS-FNA: Endoscopic ultrasonography-guided fine-needle aspiration
EUS-FNB: Endoscopic ultrasonography and fine-needle biopsy
FN: False negative
G: Grade
IQR: Interquartile range
LN: Lymph node
LN+: Lymph node metastases
M+: Distant metastases
MEN: Multiple endocrine neoplasia
MIB: Monoclonal antibody against the Ki-67 antigen
MRI: Magnetic resonance imaging
NEC: Poorly-differentiated grade 3 neuroendocrine carcinoma
NF-PNET: Non-functional pancreatic neuroendocrine tumor
NPV: Negative predictive value
Octreoscan®: ^111^In-DTPA-octreotide
PET/CT: Positron emission tomography/computed tomography
PP: Pancreatic polypeptide
PPV: Positive predictive value
SD: Standard deviation
SSTR: Somatostatin receptor
SUV_max_: Maximum standardized uptake value
VHL: Von Hippel-Lindau
WHO: World Health Organization
